# Effects of tobacco smoking during pregnancy on oxidative stress in the umbilical cord and mononuclear blood cells of neonates

**DOI:** 10.1186/s12929-014-0105-z

**Published:** 2014-12-30

**Authors:** Ednildes de Almeida Olympio Rua, Marcella Leite Porto, Jean Pierre Louzada Ramos, Breno Valentim Nogueira, Silvana dos Santos Meyrelles, Elisardo Corral Vasquez, Thiago de Melo Costa Pereira

**Affiliations:** Pharmaceutical Sciences Graduate Program, Vila Velha University (UVV), Av. Comissário José Dantas de Melo, n°21, 29102-920 Boa Vista, Vila Velha, ES Brazil; Laboratory of Translational Physiology, Health Sciences Center, Federal University of Espirito Santo, Vitoria, Brazil; Laboratory of Morphology, UFES, Health Sciences Center, Federal University of Espirito Santo, Vitoria, Brazil; Federal Institute of Education, Science and Technology (IFES), Vila Velha, ES Brazil

**Keywords:** Cigarette smoke, Cord blood, Oxidative stress, Pregnant women, Apoptosis

## Abstract

**Background:**

Although cigarette smoke is known to be a complex mixture of over 4000 substances that can lead to damage through active or passive smoking, its mechanisms and biochemical consequences in pregnancy and neonates are not yet fully understood. Therefore, in the present study, we propose to study the impact of smoking during gestation on the viability of blood mononuclear cells (MNC) from umbilical cords of newborns to assess the degree of oxidative stress and cell viability. After childbirth, the cord blood and the umbilical cord were immediately collected in public hospitals in Greater Vitoria, ES, Brazil. Flow cytometry was used to analyze the cord blood followed by biochemical and histological tests to analyze possible changes in the umbilical cord.

**Results:**

Pregnant smokers had a reduction of MNC viability from the umbilical cord (10%), an increase in the production of reactive oxygen species (ROS) and an increase in cell apoptosis (~2-fold) compared to pregnant non-smokers. In the umbilical cord, it was observed an increase of advanced oxidation protein products - AOPP (~2.5-fold) and a loss of the typical architecture and disposition of endothelial cells from the umbilical artery.

**Conclusions:**

These data suggest that maternal cigarette smoking during pregnancy (even in small amounts) may compromise the viability of MNC cells and damage the umbilical cord structure, possibly by excessive ROS bioavailability.

## Background

Maternal smoking has been considered the most important modifiable risk factor associated with adverse pregnancy outcomes [1,2]. Moreover, recent epidemiological data show that almost 20–30% of women continues to smoke during pregnancy [3,4]. This behavior causes important metabolic and biochemical changes and adaptive responses in both the fetus and the mother, resulting in an increased incidence of complications such as spontaneous abortion, placental abruption, preterm delivery, intrauterine growth restriction and stillbirth [5,6]. Although there is evidence that several tobacco metabolites can cross the placental barrier and cause both perinatal and postnatal health consequences [7-9], the causal relationship between exposure to smoking and increase in human cellular injury is not yet clearly understood [10] because the human materno-fetal tissues exposed to cigarette smoking remain poorly studied [11].

It has become evident that oxidative stress is one of the most important mechanisms involved in tobacco smoking during pregnancy [6,10,12,13]. The increase in reactive oxygen species (ROS) production from exogenous and endogenous sources results in an imbalance between the generation of oxidant species and antioxidant defenses [14-16]. Consequently, ROS in fetal structures may modify the activation of a complex array of genes involved in cell cycle signal transduction and homeostasis control, contributing to defects in endogenous stem cell repair mechanisms [17] and consequently, development of many diseases [10,18,19].

Our laboratory has evaluated ROS production by flow cytometry and biochemical analysis to understand oxidative stress-related diseases using experimental models of atherosclerosis and hypertension [16,20,21]. Therefore, it seems reasonable to use these approaches to evaluate materno-fetal tissues damaged by superoxide anion (•O_2_^−^) and hydrogen peroxide (H_2_O_2_) or to evaluate the oxidative damage to DNA or proteins due to exposure of the fetus to smoking.

Therefore, the aim of the present study was to evaluate the molecular, cellular and histological parameters that might be altered in pregnant mothers and fetuses due to maternal cigarette smoking. We hypothesize that maternal smoking might impair the viability of umbilical cord mononuclear blood cells (MNC) and might lead to further injury of other tissues such us the umbilical cord, possibly mediated by oxidative stress.

## Methods

### Patients

We recruited healthy pregnant women who were admitted to the Hospitals and Clinics of the Greater Vitória (Vitória, Vila Velha and Serra, Brazil) and who voluntarily provided written informed consent in a form that was previously approved by the Brazilian Ethical Committee for human research ‘Plataforma Brasil’ (n° 06570012000005064, 12/12/2012). Exclusion criteria were: age less than 18 years, gestational age less than 37 weeks, fetal distress (Apgar score <7 at first minute), previous infection or inflammatory conditions, amniorrhexis more than 18 hours previously and presence of infectious or inflammatory processes during pregnancy or disorders such as cardiovascular and/or renal diseases, diabetes mellitus and pre-eclampsia. Gestational age was determined by the last menstrual period and confirmed by the Capurro index after birth. None of the fetuses showed an abnormality. All subjects were of similar socio-economic status and lived in urban areas. The study population consisted of 69 healthy pregnant women. At the first visit, a history of smoking was obtained by directly questioning the pregnant women. Smokers were defined as women who self-reported a maintained smoking habit of at least one cigarette per day during pregnancy (median: 6; range: 1–20). Non-smokers were defined as women who had never smoked and were not exposed to environmental tobacco smoke during their current pregnancies.

### Blood and umbilical cord samples

Mixed venous and arterial umbilical cord blood was obtained immediately postpartum from the umbilical vein after clamping the cord. EDTA blood samples were stored at 4°C until flow cytometry analysis. Immediately after cord blood collection, each umbilical cord was divided into two sections, proximal and distal portions of the placenta, which were stored at −196°C and at 4°C, respectively, in 10% (w/v) formaldehyde.

### Cell samples and viability assay for flow cytometry

Blood samples were homogenized and mixed with Dulbecco's Modified Eagle Medium (DMEM) to nourish the cells. The homogenate was then loaded onto a Ficoll-Paque™ PLUS (GE Healthcare, Waukesha, WI) gradient. The layer containing mononuclear cells (MNCs) was removed, washed thrice with PBS and resuspended for flow cytometry analysis. Cell viability was assessed by propidium iodide (PI) staining exclusion. A total of 10^6^ cells were incubated with 2 μL of PI for 5 min in the dark at room temperature. The cells were washed with PBS and analyzed with a FACS-Canto II flow cytometer (Becton Dickinson, San Jose, CA, USA). For viability quantification, samples were acquired in triplicate, and 10,000 events were used for each measurement. Cells were excited with a wavelength of 488 nm, and PI fluorescence was detected using a 585/42 bandpass filter. Data are expressed as the percentage of unstained/viable cells [21].

### Measurement of intracellular reactive oxygen species and apoptosis

ROS was analyzed in umbilical cord blood cells by flow cytometry as previously described [21-23]. Dihydroethidium (DHE) and 2′,7′-dichlorofluorescein diacetate (DCF) were used to detect intracellular •O_2_^−^ and H_2_O_2_, respectively. Given its ability to freely permeate cell membranes, DHE has extensively been used to monitor •O_2_^−^ production. Upon reaction with •O_2_^−^, DHE is rapidly oxidized to form ethidium bromide, a red fluorescent product that intercalates between the base pairs of DNA and amplifies the red fluorescence signal. DCF is a cell-permeant indicator of H_2_O_2_ production that is nonfluorescent until oxidation occurs within the cell, converting DCF-DA into its fluorescent form, which remains trapped in the cell. DHE (160 µM) and DCF (20 mM) were added to cell suspensions (10^6^ cells), which were then incubated at 37°C for 30 min in the dark to determine the intracellular •O_2_^−^ and H_2_O_2_ concentrations, respectively [16]. Human samples that were treated with 10 μM doxorubicin or 50 mM H_2_O_2_ for 5 min to create oxidative stress without cell toxicity were used as the positive control. Cells were washed, resuspended in PBS and maintained on ice for immediate detection by flow cytometry (FACSCanto II, Becton Dickinson, San Jose, CA, USA). Data were analyzed using the FACSDiva software (Becton Dickinson), and overlay histograms were constructed using the FCS Express software. For fluorescence quantification, samples were acquired in duplicate, and 10,000 events were used for each measurement. Cells were excited with a wavelength of 488 nm, and DHE and DCF were detected using a 585/42 bandpass filter (DHE) and a 530/30 bandpass filter (DCF).

Apoptotic cells were quantified by Annexin V-FITC and Propidium iodide (PI) double staining, using an Annexin V-FITC apoptosis detection kit (Becton Dickinson, San Jose, CA, USA). In brief, cord blood-derived cells were washed twice with PBS and adjusted to 500 μL of the binding buffer (5 × 10^5^ cells). Then, 2 μl of Annexin V–FITC and 2 μl of PI were added, and the cells were gently vortexed. Cells were incubated for 15 min at room temperature (25°C) in the dark. Finally, cells were analyzed by the flow cytometer FACSCanto II (Becton Dickinson). Annexin V-/PI+ cells were recognized as unviable, Annexin V+/PI+ were considered to be in late apoptosis or necrosis and Annexin V+/PI- cells were recognized as early or primary apoptotic cells [24].

### AOPP determination

Dityrosine-containing protein cross-linking products or advanced oxidation protein products designated as AOPP, are the products of HOCl-induced chlorination of amines. AOPP were measured according to the method described by Witko-Sarsat et al. [25] using spectrophotometry with a microplate reader. Two hundred microliters of homogenate of human umbilical cord diluted 1:5 in PBS, or chloramine-T standard solutions (0 to 100 μM), were placed in each well of a 96-well microtiter plate (Becton Dickinson Labware, Lincoln Park, NJ, USA). 10 μL of 1.16 M potassium iodide (KI, Sigma) was added, followed by the addition of 20 μL of acetic acid. The absorbance of the reaction mixture was immediately read at 340 nm in a microplate reader against a blank containing 200 μl of PBS, 10 μL of KI and 20 μL of acetic acid. The chloramine-T absorbance at 340 nm was linear within the range of 0 to 100 μM. AOPP was determined when the correlation coefficient was greater than 0.95. The concentrations were expressed in μmol/mg of total protein, previously quantified by the Bradford method [26], in which samples were diluted 1:10 for dosage.

### Vessel processing and morphology

The umbilical cord samples of non-smokers (n = 10) and smokers (n = 12) were paraffin embedded, sectioned transversely (8 μm thick) and stained with hematoxylin-eosin for detection of cellularity and vascular thickness. The portion chosen was the cord halfway between the placental and fetal insertion, avoiding any areas showing physical abnormality. Images of the vein and arteries form the umbilical cord were captured with a color video camera (AxioCam ERc 5 s, Carl Zeiss, Germany) connected to a microscope (Olympus AX70, Olympus, Center Valley, PA, USA). Analysis was conducted with a Zeiss image processing software (AxioVision) by an examiner blinded to the experimental groups. The program was calibrated with a graduated slide. Using a 4× objective, the vessel cross-sectional area (VCSA) and the lumen cross-sectional area were calculated. The vessel and lumen cross sectional areas were compared between the smoking group and the non-smoking group.

### Scanning electron microscopy (SEM)

Proximal umbilical cord samples were fixed in paraformaldehyde (2%)-glutaraldehyde (2,5%) cacodylate buffer solution (0.1 M; pH 7.2) for 24 h, washed with cacodylate buffer, postfixed in a solution of 2.5% potassium ferrocyanide for 1 h, and washed again in cacodylate buffer and ultrapure water. Stereomicroscopy was used to obtain longitudinal sections of vessels from the umbilical cord samples. Sections were dehydrated in ascending grades of ethanol, subjected to critical point drying in CO_2_ (Autosandri-815, Tousimis), coated with 10 nm of pure gold in a vacuum sputter coater (Desk V, Denton Vacuum) and studied in a direct mode using a scanning electron microscope (Jeol, JEM6610 LV).

### Statistical analysis

All data are expressed as the mean ± SEM. The statistical analysis was performed using Student’s *t*-test for independent samples. The statistical analyses were performed using Prism software (Prism 6, GraphPad Software, Inc., San Diego, CA, USA). A value of p <0.05 was considered statistically significant.

## Results

### Characteristics of patients

Table 1 shows the clinical characteristics of mothers and newborns. There were no significant differences between the smoking and non-smoking groups in physical examinations, anthropometric measurements and/or conventional hematological parameters analyzed. However, we observed a remarkable decrease in the viability of blood cells of umbilical cord blood in the smokers group (86 ± 2.0%) compared to the non-smokers group (95 ± 1.1%, p <0.05.)

**Table 1:** **Patient characteristics**

**Parameters**	**Non-smokers (n = 38)**	**Smokers (n = 31)**
Age	25.8 ± 1	26.6 ± 0.9
Number of cigarettes per day	none	8.5 ± 1.1
Mean arterial pressure (mmHg)	81 ± 1.3	84 ± 1.5
Gestational age	39.8 ± 0.3	39.3 ± 0.3
Capurro index	39.8 ± 0.14	39.6 ± 0.14
Delivery		
Vaginal delivery	18/38 (47%)	27/31 (87%)
Cesarean section	20/38 (53%)	4/31 (13%)
Birth weight (g)	3248 ± 83	3030 ± 80
Apgar score (median and range) 1 min	9 (8–9)	9 (8–9)
5 min	10 (9–10)	10 (9–10)
Cephalic perimeter (cm)	33.8 ± 0.15	33.5 ± 0.16
Maternal hematocrit (%)	35.1 ± 0.53	34.5 ± 0.46
Maternal hemoglobin (g/dL)	11.8 ± 0.15	11.5 ± 0.15
Viability of umbilical cord mononuclear blood cells (%)	95 ± 1.1	86 ± 2.0*

The values are presented as means ± SEM. *p < 0.05 vs. non-smoking patients.

### Reactive oxygen species (ROS) and apoptosis in blood cells of umbilical cord blood

**Figure 1:**
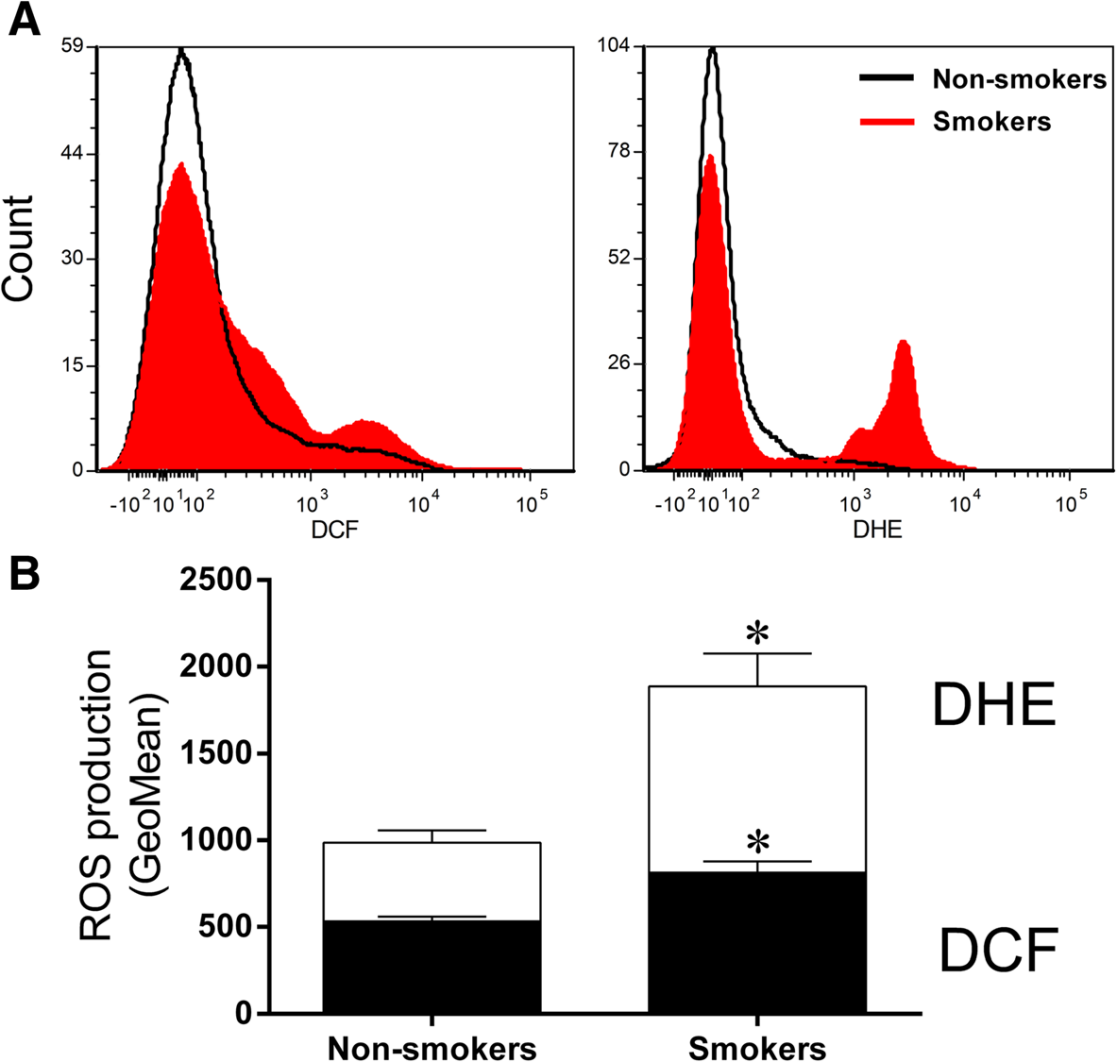
**Reactive oxygen species (ROS) production. (A)** Representative histograms from flow cytometry analysis using dihydroethidium (DHE) and 2',7'-dichlorofluorescein (DCF) in umbilical cord mononuclear blood cells from non-smokers and smokers. The log fluorescence (X-axis) illustrates the intensity of fluorescence for the number of cells counted. **(B)** Bar graph showing a remarkable increase in the level of superoxide anions (by DHE) and hydrogen peroxide (by DCF) in the smokers group (n = 23). The values are presented as means ± SEM. *p < 0.05 vs. non-smokers group (n = 30).

**Figure 2:**
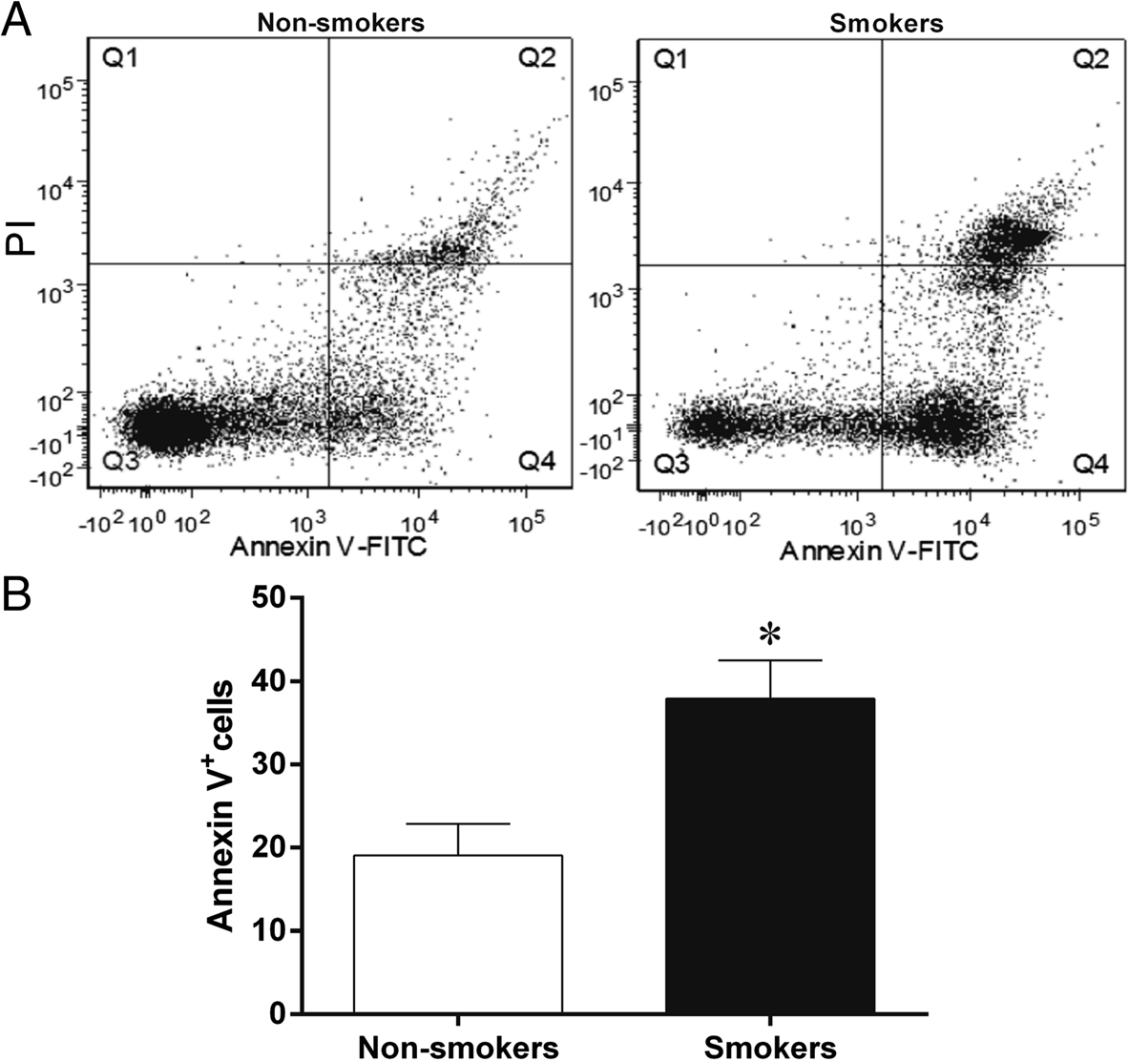
**Flow cytometry analysis of apoptosis.** Dot plots show apoptosis ratios of umbilical cord mononuclear blood cells from **(A)** non-smokers (n = 13, left panel) and smokers (n = 13, right panel) using propidium iodide (PI) and FITC-annexin V. The Q1 quadrant represents unviable cells (PI positive and annexin negative). The Q2 quadrant represents cell that are in late apoptosis or necrosis (both annexin and PI positive). The Q3 quadrant represents viable cells (both annexin and PI negative). The Q4 quadrant represents cells in early apoptosis/cell apoptosis (annexin positive and PI negative). Note the remarkable increase in apoptotic cells number (Q2 + Q4) in the smokers group (n = 13). **(B)** Bar graph shows average percentage of apoptotic cells (Q2 + Q4). The values are presented as means ± SEM. *p <0.05 vs. non-smokers.

ROS production was assessed using flow cytometry with DHE and DCF to quantify the production of •O_2_^−^ and H_2_O_2_, respectively, which are reported as geometric mean fluorescence intensity (GMFI, in a.u.). A typical histogram from flow cytometry analysis show a rightward-shift in the log of DHE and DCF fluorescence in the smokers group (Figure 1A) compared with the non-smokers group. As expected, in Figure 1B we observed a remarkable increase in the levels of ROS production in the smokers group (DHE: 1887 ± 189; DCF: 814 ± 66 a.u.) compared to the non-smokers group (DHE: 987 ± 71 and DCF: 532 ± 27 a.u., p <0.05). Apoptosis in the same cells was evaluated by flow cytometry using FITC-annexin V and PI. Figure 2 shows representative dot plots for each group. A remarkable increase in apoptotic cell number (Q2 + Q4) was observed in the smokers group (38 ± 4.5%) compared to the non-smokers group (19 ± 3.7%, p <0.05).

### AOPP determination in the umbilical cord

**Figure 3:**
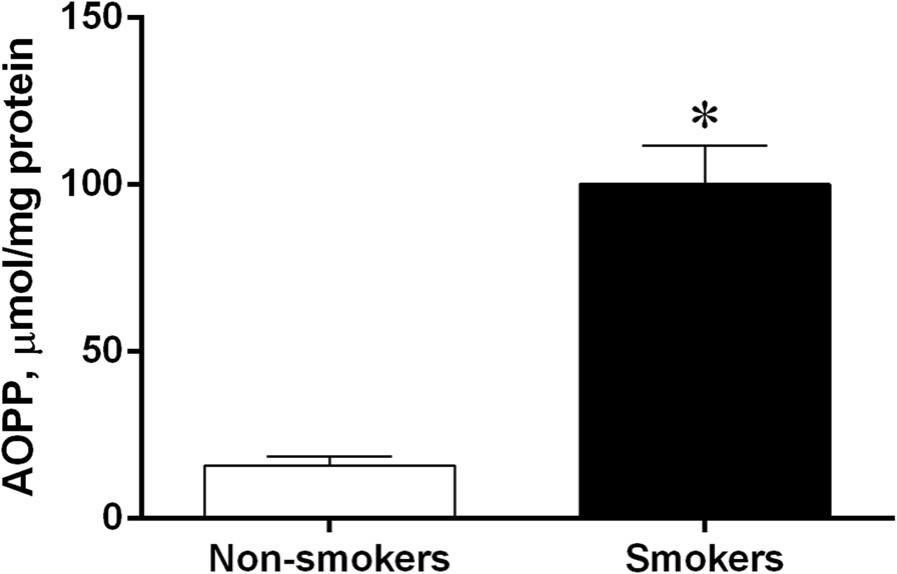
**AOPP quantification.** Levels of advanced oxidation protein products (AOPP) in umbilical cord blood from pregnant non-smokers (n = 6) and smokers (n = 8). The values are presented as means ± SEM. *p <0.05 vs. non-smokers.

To estimate the degree of oxidant-mediated protein damage in the umbilical cord, the presence of AOPP was investigated. As shown in Figure 3, AOPP levels were significantly increased (p <0.05) in the smokers group compared to the non-smokers group (232 ± 53 vs. 93 ± 14 μmol/mg protein, respectively).

### Histological examinations

**Figure 4:**
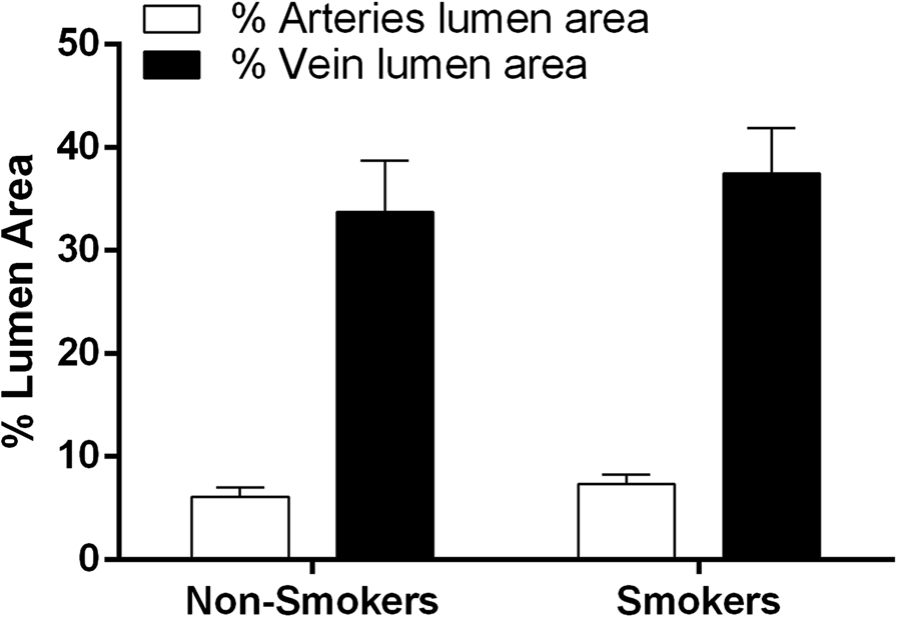
**Lumen area analysis.** Bar graphs showing percentage of cross-sectional luminal areas of arteries (white bars) and veins (black bars) from the umbilical cord of the non-smokers group (n = 10) and the smokers group (n = 12). The values are presented as means ± SEM.

**Figure 5:**
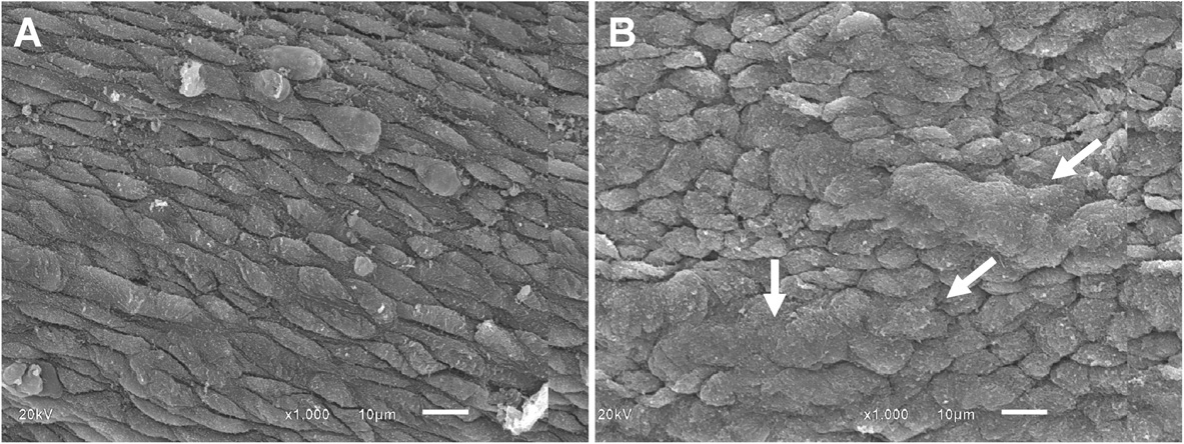
**Scanning electron microscope (SEM) photographs of the vascular endothelium from umbilical arteries of pregnant patients. A)** A typical image from a non-smoking patient shows a regular surface accompanying the longitudinal direction of the vessel. **B)** A typical image from a smoking patient shows diffuse areas of endothelial thickening with loss of the typical architecture and disposition of endothelial cells (white arrows). Scale bar: 10 µm.

Figure 4 summarizes the data of the cross sectional area of the umbilical cord vessels, showing a similar lumen area in the smokers and non-smokers groups, both in veins (37.5 ± 4.5 vs. 33.8 ± 4.9%, respectively) and arteries (7.3 ± 0.96 vs. 6.1 ± 0.92%, respectively). Moreover, we did not find significant modification of vessel total area (data not shown), indicating absence of outward vascular remodeling. Figure 5 shows typical photomicrographs of scanning electron microscopy (SEM) of vascular endothelium from umbilical arteries of pregnant patients, exhibiting diffuse areas of endothelial thickening with loss of the typical architecture and disposition of endothelial cells in the smokers group.

## Discussion

Cumulative evidence shows that cigarettes may be the single most significant source of toxic chemical exposure and chemically mediated illness in humans, and that maternal smoke exposure in pregnancy can cause substantial harm to women and their developing fetuses [27-29]. However, the causal relationship between exposure to smoke and increase in human cellular injury has not yet been clearly demonstrated [10]. Therefore, it is important to determinate the impact and etiology of tobacco-related effects on the maternal-fetal interface throughout pregnancy. In the present study, we report for the first time that exposure to cigarette smoke may compromise the cell viability of MNC and damage the umbilical cord structure, possibly mediated by oxidative stress.

In the present study, we evaluated two groups of patients with similar characteristics appropriate for normal gestational age who had not been exposed to any adverse events (other than cigarette smoke, for the one group). Our results on cigarette smoke exposure highlight two important findings: First, the biochemical and structural alterations observed were not changed by previous factors intrinsic to smokers (e.g., malnutrition, anemia or arterial hypertension). Second, our data revealed that smoking (even in small amounts) may compromise the neonate in parameters not conventionally analyzed in previous clinical studies, such as impaired umbilical cord blood MNC viability.

We have previously demonstrated the applicability of flow cytometry with DHE and DCF to evaluate the production of principal ROS (•O_2_^−^ and H_2_O_2_) in mouse models of various diseases [16,20,21,23]. In this study, we extended this analysis to human tissues by using flow cytometry and found a higher level of ROS production in umbilical cord blood MNC from smoking patients. This finding could indicate that macromolecular damages might occur in parallel by modifying intracellular calcium homeostasis and several metabolic pathways leading to apoptotic cell death, which has been shown in other ROS-related diseases [16,18,21]. The annexinV/propidium iodide staining approach allowed us to confirm our hypothesis of increased apoptosis of umbilical cord blood MNC from smoking patients. Our finding corroborates the idea from other research that there are cellular impairments both in cord blood by gene expression profile [30] and in neonatal lymphocytes by cytogenetic analyses [4].

Although the apoptotic damage observed in MNC might not accurately reflect similar injury in other tissues, the early prejudice in this cell group could have an important clinical applicability. First because MNC play a central role in the development and repair of damaged tissues throughout the life cycle, a compromise in MNC might contribute to the early generation of many diseases in childhood, including cancer [4,31], cardiovascular disease [32], respiratory disease [33], infectious disease [34] and/or mental disease [35]. Second, the human umbilical cord blood MNC, which contain hematopoietic, mesenchymal and endothelial stem cells [36], have a naive immunologic phenotype and a high regenerative potential, making them a preferable choice for autologous transplantation in recent therapeutic strategies for cancer [37], neuroregeneration [38], hematopoietic disease [39] and/or cardiovascular disease [40,41]. Therefore, our data indicate that pregnancy-related factors such as exposure to cigarette smoke may compromise the success of cell therapy, in addition to technical approaches such as cryopreservation or thawing [42,43].

We also found strong evidence of oxidative stress in the umbilical cord of smoking patients by investigating AOPP. Proteins are likely to be major targets of ROS, as a result of their abundance in cells (approximately 70% of the dry mass of most cells) and most tissues and their rapid rates of reaction with many radicals and with other oxidants. ROS attack against proteins can modify proline and basic amino acid residues, generating carbonyl moieties, protein-protein cross-linkages, and oxidation of the protein backbone, resulting in protein fragmentation and loss in specific function [44]. An advantage of AOPP analysis is due to the relatively long half-life of such oxidized proteins [44], which can better demonstrate the cumulative effect of oxidative stress. Moreover, the AOPP remain stable during sample storage both at −20°C and −80°C for 6 months [45], facilitating the subsequent analysis of the collected samples, which are not collected simultaneously. It should be noted that our data corroborate the data of several other groups that have successfully demonstrated that AOPP is a potentially useful clinical marker for the prediction of the stress/damage process and age-related morphological and physiological disturbances [19,25,44,46].

Anatomopathological studies of umbilical cords and placentas of newborn infants from mothers who are smokers demonstrate that tobacco increases the release of vasoactive substances and decreases the release of vasodilator substances such as nitric oxide (NO) [47,48], contributing further to oxidative stress, shear stress, hypoxia and inflammation [13,49]. Based on a large number of studies in animal models, these four types of stimuli can drive a compensatory adaptive structural change known as outward vascular remodeling [50-52]. Therefore, we analyzed whether the umbilical arteries and/or vein developed this alteration. For the first time, we have shown that this possible compensatory mechanism does not appear to occur in the umbilical cords of pregnant smokers.

The endothelium of umbilical blood vessels (both vein and arteries) consists of a single layer of endothelial cells whose cytoplasm is rich in mitochondria, the main source of ROS [53]. While low levels of ROS are essential and participate in important intracellular signaling pathways, excessive generation of ROS may result in endothelial damage and plays a critical role in the progressive deterioration of vessel structure and function [54,55]. Although this concept is well-established in experimental models [16,54] and in some classes of patients [56-58], few studies have been conducted in newborns of cigarette smoking mothers [59,60]. In this study, our data indicated an increase of the oxidative stress biomarker AOPP in the umbilical cord and loss of the typical architecture of endothelial cells in the same tissue (by SEM analysis), providing further evidence of ROS-mediated endothelial damage in neonatal passive smokers. In bone marrow-derived MNC, there exists a population of endothelial progenitor cells thought to engage in endothelial repair that are considered potential therapeutic agents in many pathological conditions [61,62]. Recent studies have reported that the disequilibrium between reparative endothelial cells and inflammatory leukocytes may compromise the balance of vascular injury and repair [16,61,63]. Our data are compatible with this hypothesis, because the compromise of MNC in smoking mothers could in part explain the impaired endothelial repair capacity, thereby contributing to endothelial dysfunction.

A relative limitation of our study is that we did not analyze these parameters with cotinine serum/urine/cord blood determination. Cotinine has a longer half-life than nicotine, and cotinine concentrations in serum, urine, hair, and saliva are commonly used as biomarkers of recent tobacco exposure in epidemiological studies [9,64]. There is most likely no placental barrier for plasma cotinine between pregnant mothers and their newborns. Lack of a placental barrier for cotinine (and probably nicotine) can partially explain smoking-related perinatal disorders [4,9]. Therefore, combining the maternal self-report of smoking with the level of urine cotinine concentration could improve the precision of the exposure estimates [8,27,65].

## Conclusions

These data show that maternal cigarette smoking during pregnancy may compromise the viability of MNC and damage the umbilical cord structure, possibly by excessive ROS bioavailability. These results may provide a new direction and alternative approach to investigations of the impact of cigarette smoking during pregnancy.
